# Expression of the T Cell Receptor αβ on a CD123+ BDCA2+ HLA-DR+ Subpopulation in Head and Neck Squamous Cell Carcinoma

**DOI:** 10.1371/journal.pone.0015997

**Published:** 2011-01-11

**Authors:** Annette Thiel, Rebecca Kesselring, Ralph Pries, Alexander Puzik, Nadine Wittkopf, Barbara Wollenberg

**Affiliations:** 1 Department of Otorhinolaryngology, University of Schleswig-Holstein Campus Lübeck, Lübeck, Germany; 2 Department of Pediatrics, University of Freiburg, Freiburg, Germany; Karolinska Institutet, Sweden

## Abstract

Human Plasmacytoid Dendritic Cells (PDCs) infiltrating solid tumor tissues and draining lymph nodes of Head and Neck Squamous Cell Carcinoma (HNSCC) show an impaired immune response. In addition to an attenuated secretion of IFN-α little is known about other HNSCC-induced functional alterations in PDCs. Particular objectives in this project were to gain new insights regarding tumor-induced phenotypical and functional alterations in the PDC population. We showed by FACS analysis and RT-PCR that HNSCC orchestrates an as yet unknown subpopulation exhibiting functional autonomy in-vitro and in-vivo besides bearing phenotypical resemblance to PDCs and T cells. A subset, positive for the PDC markers CD123, BDCA-2, HLA-DR and the T cell receptor αβ (TCR-αβ) was significantly induced subsequent to stimulation with HNSCC in-vitro (p = 0.009) and also present in metastatic lymph nodes in-vivo. This subgroup could be functionally distinguished due to an enhanced production of IL-2 (p = 0.02), IL-6 (p = 0.0007) and TGF-β (not significant). Furthermore, after exposure to HNSCC cells, mRNA levels revealed a D-J-beta rearrangement of the TCR-beta chain besides a strong enhancement of the CD3ε chain in the PDC population. Our data indicate an interface between the PDC and T cell lineage. These findings will improve our understanding of phenotypical and functional intricacies concerning the very heterogeneous PDC population in-vivo.

## Introduction

Dendritic cells (DCs) are bone marrow derived antigen presenting cells, such as B cells and monocytes and appear to be indispensable for initiating appropriate immune responses. A complicated trafficking system leads them from the bone marrow through the bloodstream to distinct peripheral tissues, where different immature DC populations reveal unique kinds of chemokine responsiveness [1]. Finally they migrate to lymphoid organs in order to present processed antigens to lymphocytes and to stimulate immune responses [2]. Two main human DC subsets are to be distinguished: myeloid dendritic cells (MDCs) including Langerhans cells, dermal dendritic cells and interstitial DCs versus plasmacytoid dendritic cells (PDCs) [3]. Referring to their plasma cell-like appearance PDCs were first identified as ‘Plasmacytoid monocytes/T cells’ in T cell areas of lymphoid organs, later also in peripheral blood and tonsils [4].

PDCs are to a small extent capable of presenting antigens to T cells, however their main known function is characterized by the ability to produce large amounts of IFN-α in viral infections in-vivo. In-vitro, IFN-α secretion is also triggered by CpG-oligonucleotides, which consist of unmethylated CpG-dinucleotides embedded in a certain sequence context within bacterial DNA [5], [6], [7]. Recently, different PDC subsets with distinct phenotype und functions have been identified [8], [9].

It has been shown that human solid tumor tissues of Head and Neck Squamous Cell Carcinoma (HNSCC) are infiltrated by PDCs, which seem to intensely compromise proper immune functions in this environment [10]. Cells of head and neck cancer are known to develop molecular strategies to escape from efficient anti-tumor immune responses. Up to now it was assumed that mainly production of various immunosuppressive mediators by tumor cells contributes to a heavily deranged immune defence [11].

However, the molecular mechanisms responsible for these immunomodulatory transformation processes and the biosynthesis of the immunosuppressive HNSCC microenvironment remain almost unknown [12], [13]. In this work we present a new approach to the subject by introducing a novel, functionally distinct subpopulation bearing phenotypical resemblance to PDCs and T cells in HNSCC.

## Results

### Phenotypical alterations in the PDC population subsequent to incubation with HNSCC cell-lines in-vitro were narrowed to incomplete maturation and up-regulation of the receptors CD4 and CD45RA

**Figure 1:**
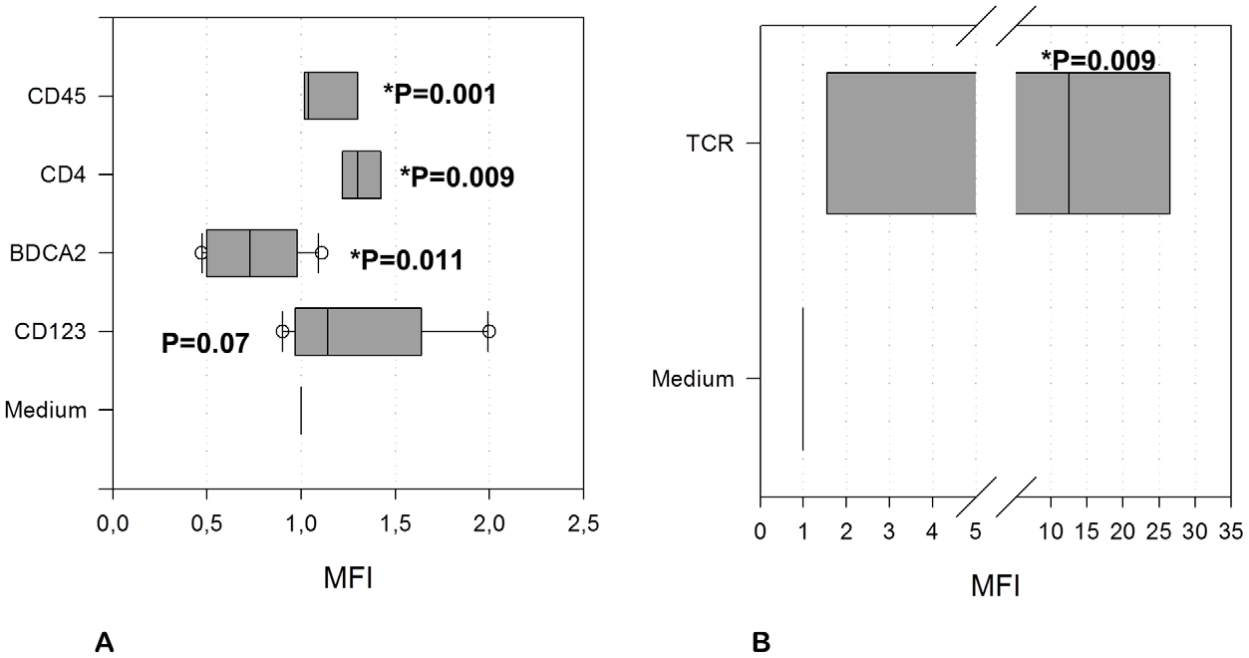
Incomplete maturation in the PDC population and induction of the TCR-αβ positive subset post stimulation. **A** CD123+ HLA-DR+ BDCA-2+ cells showed an incomplete maturation subsequent to stimulation with HNSCC. BDCA-2 was down-regulated (p = 0.011) while CD123 was up-regulated (p = 0.07). Furthermore, a highly significant up-regulation of CD4 (p = 0.009) and CD45RA (p = 0.001) was observed. **B** TCR-αβ positive, CD123+ HLA-DR+ BDCA-2+ cells were significantly (p = 0.009) induced subsequent to stimulation with HNSCC cell-lines for 12 hours. The Mean Fluorescence Intensity (MFI) was determined separately for each Isotype control and each subpopulation and then subtracted from the positive results before calculating the relative MFI. Figure 1 shows the relative MFI after incubation with HNSCC in relation to the medium control MFI. The control MFI is equal to 1 in each case by definition. The data are presented in box plots, showing the median, the lower and the upper quartile.

Classical PDC maturation is phenotypically defined by the up-regulation of CD123, CD80, CD83, CD86 and HLA-DR in addition to the down-regulation of the C-type Lectin BDCA-2 [14], [15], [16]. However, subsequent to incubation with permanent HNSCC cell-lines for 12 hours, PDCs show only a partial maturation. Unlike classical maturation, HNSCC has neither any impact on HLA-DR nor on the co-receptors CD80, CD83 and CD86. BDCA-2 was significantly down-regulated (p = 0.01), while CD123 was up-regulated (p = 0.07). In some donors HLA-DR was strongly down-regulated. Furthermore, a highly significant surface elevation of CD4 (p = 0.009) and CD45RA (p = 0.001) was observed (Figure 1A).

All other analysed receptors, which were lineage markers (CD3, CD14, CD16, CD19, CD20, CD56), Fc-receptors (CD16, CD23, CD32, CD64), further T cell markers (CD1a, CD2, CD3, CD5, CD8, CD25, GITR, CTLA-4), activation markers (CD40, CD69), stem cell markers (CD34, CD117, CD133) and the myeloid marker CD11c, were not significantly affected by tumor cell-lines in the classical PDC population (data not shown).

### HNSCC induced a TCR-αβ positive CD123+ BDCA2+ HLADR+ subset

**Figure 2:**
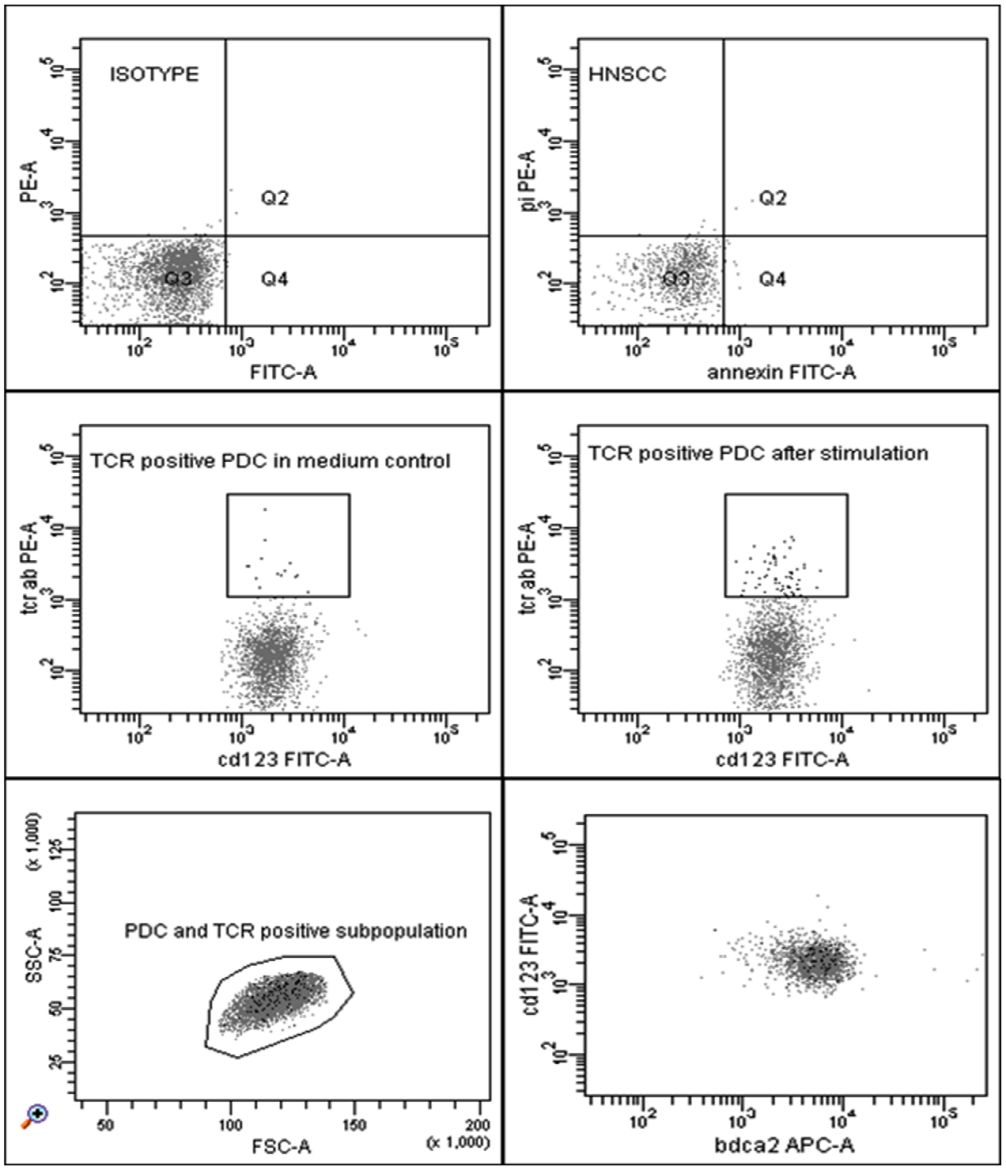
FACS plots showing the TCR-αβ positive subset subsequent to stimulation with HNSCC. An IgM-Isotype control was used as a negative control. Cell viability was confirmed by staining with Annexin and PI. TCR-αβ positive cells were significantly (p = 0.009) enhanced from 1.6% (mean) +/−1.5% (SD) in the medium control to 4.5% (mean) +/−0.88% (SD) in healthy donors after stimulation with tumor in-vitro. The TCR positive subset expressed the surface markers CD123, HLA-DR (not shown) and BDCA2 and was located in the centre of the PDC population when back gated to FSC/SSC. The black dots represent the TCR-αβ positive cells in the lower scattergrams.

**Figure 3:**
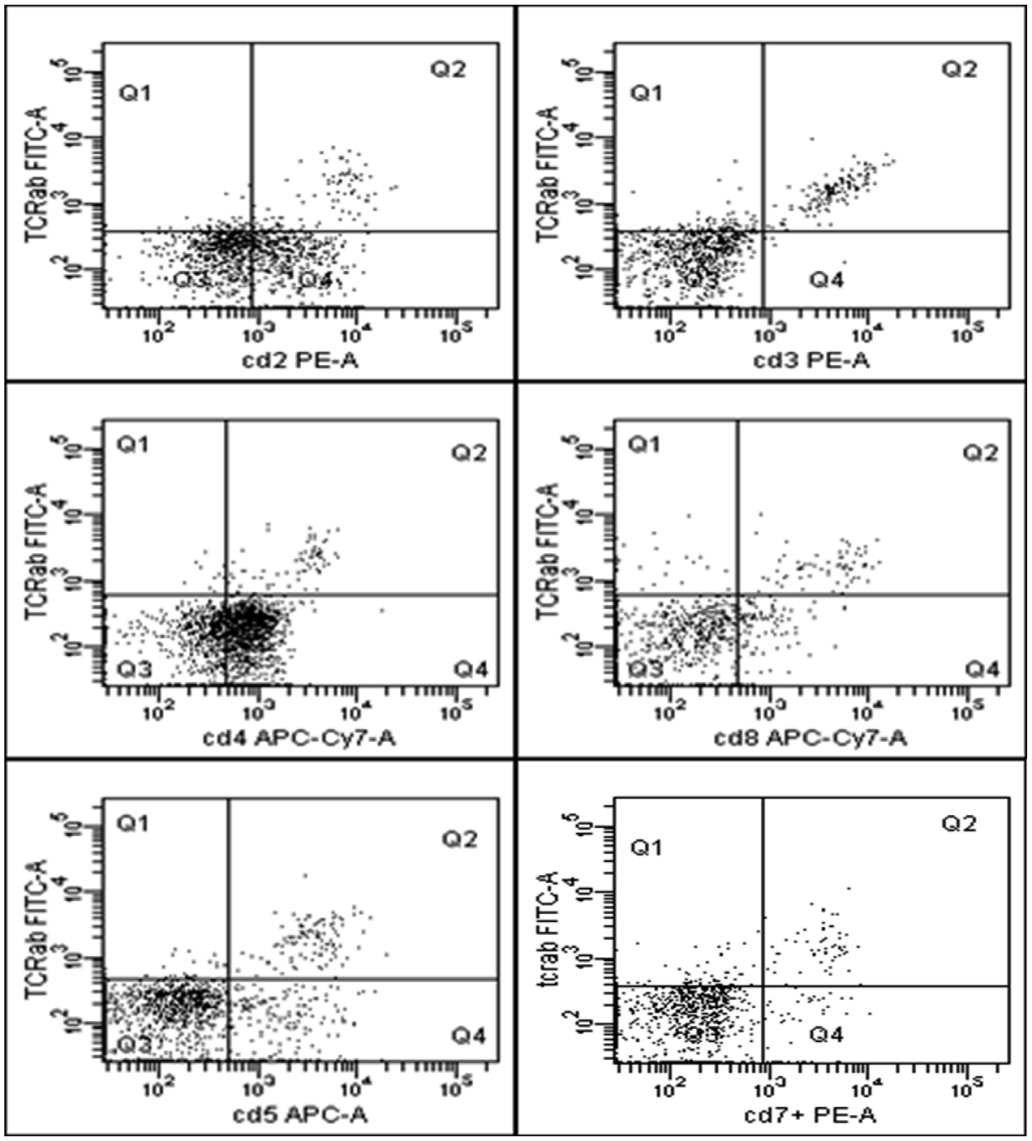
Surface marker expression on the TCR-αβ+ cells. The TCR-αβ positive subset expressed the surface markers CD2, CD3, CD4, CD5, CD7 and CD8.

HNSCC induced a TCR-αβ positive CD123+ BDCA2+ HLADR+ subset. This subset was significantly (p = 0.009) up-regulated subsequent to stimulation with HNSCC cell-lines for 12 hours and enhanced from 1.6% (mean) +/−1.5% (SD) in the medium control to 4.5% (mean) +/−0.88% (SD) after stimulation (Figure 1B). Furthermore it was located in the centre of the PDC population when back-gated to the FACS forwards/sidewards scatter (Figure 2) and exhibited a double-positive phenotype for CD4 and CD8 in-vitro, however, partly a double-negative phenotype in-vivo (data not shown). Furthermore, this subset was positive for CD2, CD3, CD5 and CD7 (Figure 3). CD1a, CTLA-4, FOXP3, CD34 and CD117 were not expressed. Some donors revealed CD25dim+/TCR-αβ+ cells, however we could not show any significance for the expression of the surface marker CD25. CD25 positive cells were likewise located in the PDC region (SSC/FSC) and represented larger cells than the remaining TCR-αβ positive subset (Text S1). Doublets were excluded by gating FSC-H against FSC-A. The TCR-γ/δ could not be detected.

Furthermore a RT-PCR was performed subsequent to stimulating and cell-sorting the PDC population. Compared to the medium control, a distinct up-regulation of CD3ε and the pre-TCRα mRNA was detected after incubation with HNSCC. CD3γ and CD3δ mRNA could not be detected. As T cells do express CD3γ and CD3δ mRNA, these lanes were used as a control in order to exclude a contamination by ordinary T cells.

**Figure 4:**
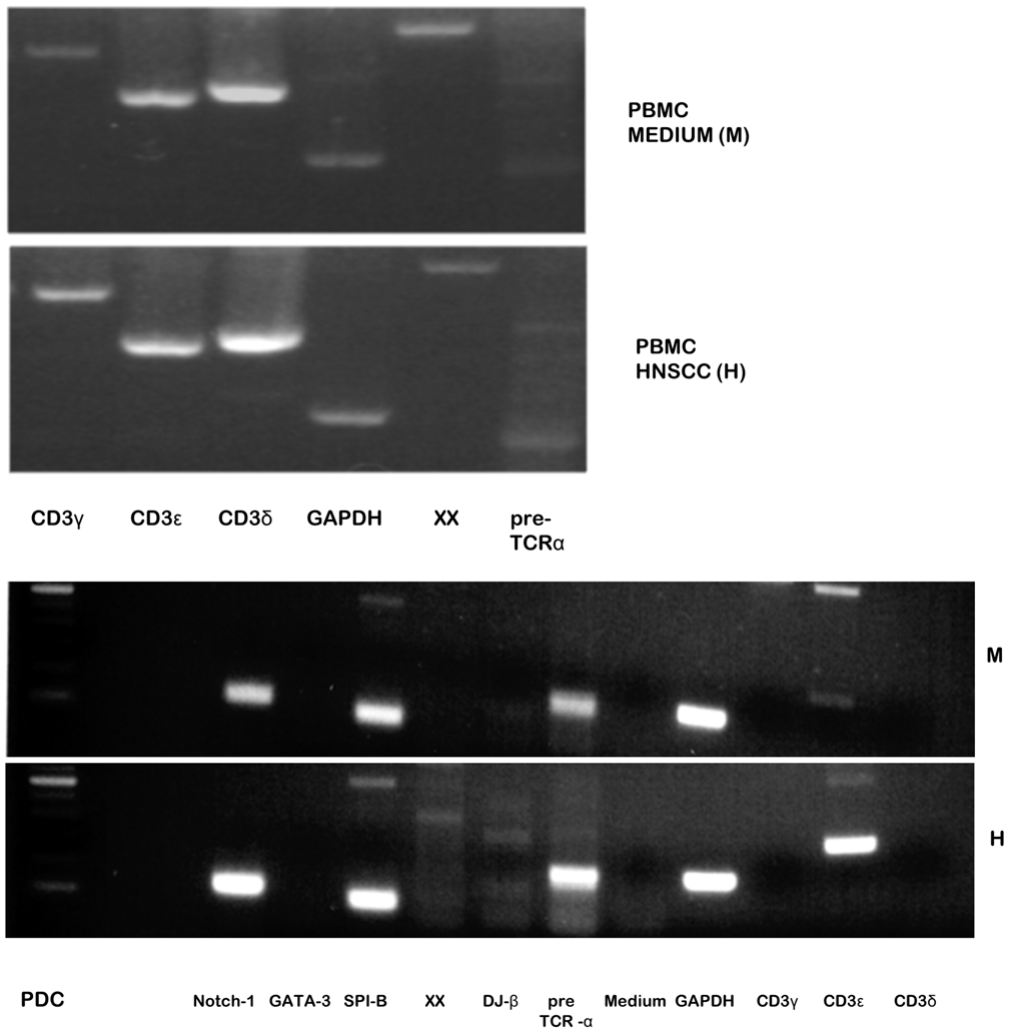
Impact of HNSCC on mRNA-levels in the PDC population. We demonstrated an increase of the pre-TCRα-receptor mRNA-levels besides a strong enhancement of the CD3ε chain mRNA-levels after stimulation with HNSCC in the PDC population. Both, the CD3δ and γ chain were not detectable. In order to prove recombination of the TCR-β chain in the PDC population, we also analysed mRNA up-regulation for D-J-β, the earliest point of recombination of the TCR-β chain. Indeed, after stimulation with tumor we did detect D-J-β mRNA. For a positive control PDC depleted PBMCs were used in order to demonstrate the presence of CD3δ and γ mRNA in contrast to the isolated CD123+ HLA-DR+ BDCA-2+ population. The pre-TCRα mRNA could hardly be detected.

Furthermore the data comprise a strong indication for the up-regulation of the recombined D-J-β chain in the PDC population subsequent to stimulation with HNSCC cell-lines (Figure 4).

### The TCR-αβ positive CD123+ HLA-DR+ BDCA-2+ cells revealed distinct functional properties in-vitro and in-vivo

**Figure 5:**
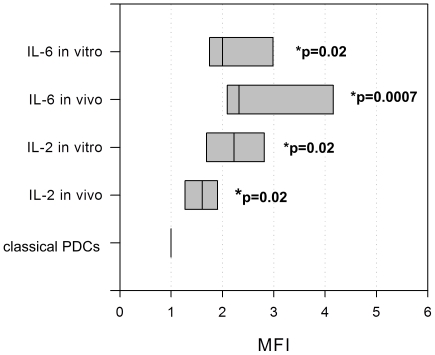
Cytokine production in the TCR-αβ positive subset. The TCR-αβ positive cells produced higher levels of IL-2 and IL-6 when compared to the TCR-αβ negative CD123+ HLA-DR+ BDCA-2+ cells. All results were significant (p = 0.02), a dominant IL-6 secretion (p = 0.0007) was highly significant for the TCR-αβ positive cells in-vivo. The Mean Fluorescence Intensity (MFI) was determined separately for each Isotype control and each subpopulation and then subtracted from the positive results before calculating the relative MFI. Figure 5 shows the relative MFI of the TCR-αβ positive subset in relation to the MFI of the classical PDC population. The MFI of the classical PDC population is equal to 1 in each case by definition. The data are presented in box plots, showing the median, the lower and the upper quartile.

**Figure 6:**
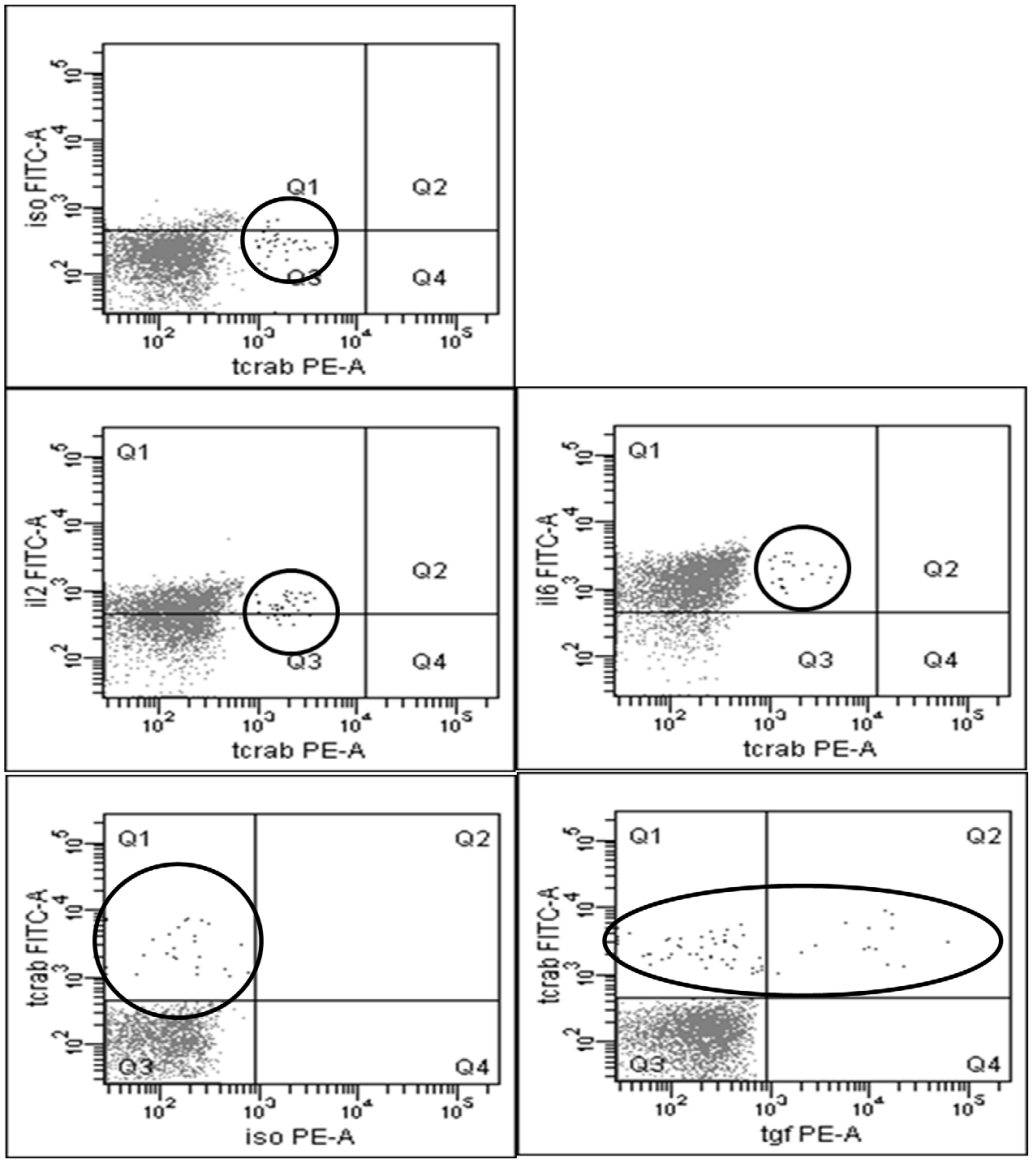
FACS plots showing the cytokine production in the TCR-αβ positive and negative subset. FACS plots showing IL-2 and IL-6 production in-vivo. The black circles identify the TCR-αβ positive population. In this sample, the TCR-αβ positive cells comprised 2% of the PDC population and partly produced TGF-β. TGF-β secretion was analysed four times.

We analysed the cytokine production (IL-2, IL-4, IL-6, IL-8, IL-10 and TGF-β) of the TCR-αβ positive subset in-vitro and in single cell suspensions of metastatic lymph nodes from HNSCC patients. The results were compared to the cytokine production of the TCR-αβ negative PDC population. We were able to significantly demonstrate a higher intracellular production of IL-2 and IL-6 in the TCR-αβ positive PDC population in-vitro and in-vivo (p = 0.02). A dominant IL-6 secretion was highly significant (p = 0.0007) for the TCR-αβ positive cells in-vivo (Figure 5 and 6). 90–100% of the TCR-αβ+ cells were positive for IL-2 and 100% of the TCR-αβ+ cells were positive for IL-6 in each sample (p<0.001). The classical PDCs do not confidently produce IL-2. The FACS plots show a shift in the population, however no circumscribable positive subset. About 80% of the classical PDCs were positive for IL-6 (p<0.001).

### Expansion of the TCR-αβ subset could not be demonstrated

**Figure 7:**
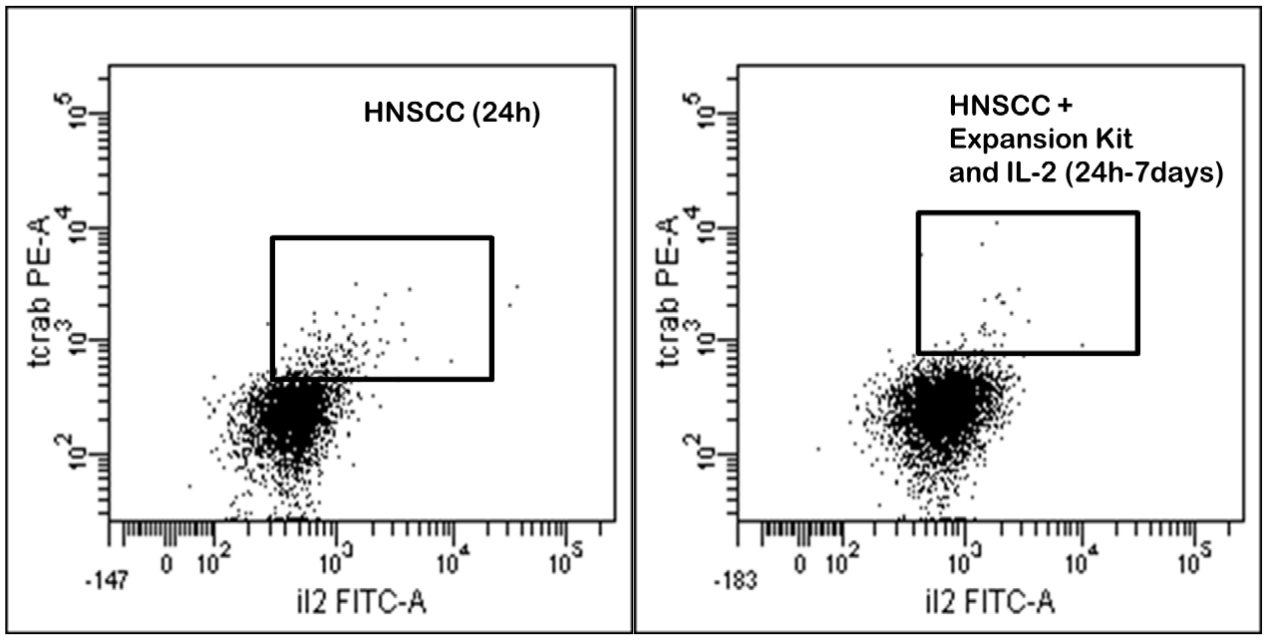
We incubated isolated CD123+ BDCA-2+ HLA-DR+ cells with HNSCC and the T cell Expansion Kit (Miltenyi) in addition to IL-2 (20-200 U/l) for 3-7 days. Less TCR-αβ+ cells were revealed (after 1-7 days) when compared to the control with HNSCC after 24 h. Presumably this can be attributed to the down-regulation of the TCR subsequent to cell activation.

Expansion of the TCR-αβ+ subset could not be assured, as the TCR-αβ was barely detectable after cross-linking CD3/28. Nevertheless, we incubated isolated CD123+ BDCA-2+ HLA-DR+ cells with HNSCC and the T cell Expansion Kit (Miltenyi) in addition to IL-2 (20–200 U/l) for 3–7 days. Less TCR-αβ+ cells were revealed (after 1–7 days) when compared to the control with HNSCC after 24 h (Figure 7). Presumably this can be attributed to the down-regulation of the TCR subsequent to cell activation. However, the CD123+ BDCA-2+ HLA-DR+ cells, which usually do not survive longer than 12–24 hours in-vitro, survived up to 7 days subsequent to stimulation with the Expansion Kit and IL-2. Incubation with IL-4 (1–10 pg/ml), IL-6 (500–4000 pg/ml), IL-8 (500–1000 pg/ml), IL-10 (10 ng/ml) and TGF-β (10 ng/ml) did not lead to any significant expansion of the subset. Cytokine concentrations were chosen according to the cytokine secretion of the HNSCC cell lines, which induced the TCR-αβ+ subset [17].

### The transcription factor Notch-1 is up-regulated in the PDC population subsequent to incubation with HNSCC cell-lines

As the transcription factors Notch-1, GATA-3 and SPI-B have been shown to control the T cell/PDC lineage decision in early thymocytes [18], the mRNA levels were analysed in the PDC population after stimulation with HNSCC. Indeed, we found elevated levels of Notch-1 mRNA, however no up-regulation of GATA-3 and SPI-B mRNA could be detected (Figure 4).

## Discussion

Numerous pro-inflammatory, pro-angiogenetic and immuno-suppressive cytokines [11], [17], [19], [20] within the HNSCC microenvironment and in the serum of tumor patients are suspected of benefiting tumor growth and metastasis besides severely impairing the function of various immune cells [21], [22], [23]. Especially IL-6, correlating with tumor stage and lymph node status [24], has been proven to be an independent predictor of poor survival [25]. Solid HNSCC and metastatic lymph nodes have been found to be infiltrated by various species of immune cells including PDCs, which are supposed to participate in the formation of the HNSCC microenvironment due to functional alterations [10], [26], [27].

### PDCs in HNSCC

In accordance with the postulation above, we observed functionally disabled PDCs after stimulation with tumor. As already revealed in non-small cell lung cancer [28] we concordantly demonstrated an impaired phenotypical maturation in the PDC population in-vivo and after stimulation with HNSCC cell-lines for 12 hours in-vitro.

Consistent with normal maturation CCR7 [29] and CD123 are up-regulated whilst BDCA2 is down-regulated. In contrast the co-stimulatory receptors (CD80, CD86) and CD83 are not influenced by the tumor, HLA-DR expression is even decreased in many cases and the early activation marker CD25 [30] is only presented by a small subpopulation [unpublished data]. As we have already shown in earlier studies, the immature phenotype also correlates with a disturbed functional maturation. PDCs stimulated with CpG-2216 reveal an impaired IFN-α production in the HNSCC environment [29].

### TCR-αβ positive CD123+ HLA-DR+ BDCA-2+ cells in HNSCC

First of all the discovery of the TCR-αβ on a subpopulation of CD123+ HLA-DR+ BDCA-2+ cells confronted us with two main questions: did we really find a new population expressing the TCR and not activated, blastic T cells similar to the recently characterised CD4+ Th-APC [31]? Furthermore, as Busch et al. [32] discovered the transfer of T cell surface molecules to DCs upon CD4+ T cell priming: did we observe a TCR exchange or production?

Therefore we performed a RT-PCR for the earliest point of recombination: the DJ-β chain, for the CD3 chains and the pre-TCRα. After stimulation with HNSCC cell-lines, a weak induction of the DJ-β chain was exhibited whilst no trace was visible in the medium control. The other striking point is the difference in the distribution of the CD3 chains and the pre-TCRα in the PDC population compared to the PBMC control. Whilst the lymphocytes in the PBMC control express the CD3δ, ε and γ chain mRNA, the PDC population only exhibits a weak expression of the CD3ε chain which is clearly enhanced after stimulation. Similar results are demonstrated for the pre-TCRα mRNA. These findings indicate an intracellular production of parts of the T cell receptor and exclude a contamination by ordinary T cells.

### Cytokine expression

Due to the size of the TCR positive subpopulation, the FACS facility was not able to reach a high purity by cell sorting this subset, so we had to reduce functional analyses to the FACS. Currently we are working on expanding cocktails for this subpopulation.

By FACS analysis we were able to demonstrate significantly higher amounts of intracellular IL-2 and IL-6 for the TCR positive subpopulation. Especially production of IL-6 was highly significant in-vivo. Furthermore, the production of TGF-β was observed in the TCR positive subset. Therefore, we postulate a role for this population in tumor growth, metastasis and induction of tolerance. IL-6 and likewise CD25/HLA-DR+ T cells, which are at least phenotypically associated with the new subpopulation, have been proven to be a predictor of poor survival in HNSCC [25], [33]. Additionally, IL-6 promotes growth, survival, invasiveness and also secretion of IL-6 in different tumors in-vivo [34], [35], [36]. Furthermore, also the secretion of TGF-β has been linked to epithelial-mesenchymal transition, metastasis and the induction of oncogenic properties [37], [38], [39] besides impairing the secretion of IFN-α in PDCs [40]. Doubtless, these postulations are speculative at the time. However, as Baban et al. and Matsui et al. [8], [9] have already demonstrated functionally distinct PDC subsets, we consider it possible that this minor subset has a relevant impact on HNSCC.

### Origin of the subpopulation

A further question concerns the cell lineage of this subpopulation. The TCR positive CD123+ HLA-DR+ BDCA-2+ cells may originate from a T cell subpopulation exhibiting PDC features besides lacking the CD3 δ and γ chain. Likewise they may originate from a subpopulation of PDCs. Similar to other recently discovered populations showing an interface between two lineages [41], [42], a determination of the lineage will become a major challenge, as a mutual contamination cannot be absolutely excluded during isolation. A possible approach could include the detection of IFN-α and the transcription factor E2-2 [43].

As NOTCH-1 has been shown to regulate PDC versus T cell lineage decision through control of GATA-3 and SPI-B [18] we analysed the mRNA levels of the PDC population subsequent to stimulation. However, although NOTCH-1 mRNA was up-regulated after exposure to HNSCC cell-lines, we could not demonstrate any expression of GATA-3. On the contrary, the levels of SPI-B, which is supposed to inhibit T-, NK- and B cell development [44], were actually slightly elevated. In this respect we were able to exclude the relevance of this pathway for the TCR positive subpopulation.

Besides expanding the new subset for functional analysis versus demonstrating their relevance as non-proliferating bystander T cells [45], determination of the lineage and functional analysis of the TCR signalling, in-vivo models will have to clarify the relevance of this population in HNSCC.

## Materials and Methods

### Ethic statements

An ethic proposal concerning the abstraction of tumor tissue during operations of tumor patients was approved by the ethics committee, Campus Lübeck. All experiments have been conducted according to the principles expressed in the Declaration of Helsinki. Written informed consent was obtained in each case before abstracting tissue and blood (Text S2, S3, S4).

### Isolation of PDCs

For in-vitro studies PDCs were isolated from human peripheral blood provided by the blood bank of the University Hospital Lübeck, Germany. Blood donors were 18–65 year-old healthy donors who were tested to be negative for allergies. Additional exclusion-criteria were manifest infections during the last 4 weeks, fever or medication of any kind. Peripheral mononuclear blood cells (PBMCs) were obtained from buffy coats by Ficoll-Hypaque density gradient centrifugation as described elsewhere [46]. PDCs were isolated by magnetic bead separation using magnetic labelled anti-BDCA-4 antibodies (Miltenyi, Bergisch Gladbach, Germany).

### Single-cell-suspensions

After removal of fat, blood or necrotic areas, tumor draining lymph nodes were washed in phosphate buffered saline (PBS), cut into little pieces in a Petri dish covered with PBS and washed again with PBS by gravity sedimentation in a 50 ml conical centrifuge tube. The tissue pieces were then covered with 31,5 mg Collagenase/ml PBS and 3,99 mg/ml Hyaloronidase/ml PBS in DMEM and incubated in a 37°C water bath for 30–120 minutes. Digestion was checked every 30 minutes. In a further step 33,4 mg Dispase/ml PBS was added for 30–60 minutes. Brefeldin A (eBioscience, San Diego, USA) was supplied during all steps. The digest was then passed through a 100 µm nylon mesh in order to remove clumps and washed several times.

### Cell cultures

The permanent HNSCC cell lines BHY (DSMZ Germany), PCI-1 and PCI-13 (generously provided by Prof. T. Whiteside, Pittsburgh Cancer Institute) were cultured in Dulbecco’s Modified Eagle Medium (DMEM, Gibco, New York, USA) supplemented with 10% FCS, 1 mM glutamine, and 0.1 mM sodium pyruvate. All compounds were purchased Endotoxin tested.

### Incubation

Depending on the amount of cells, PDCs were incubated with HNSCC supernatants or co-cultured with the tumor cell-lines BHY, PCI-1 and PCI-13 at a concentration of 480000cells/60 µl in U-shaped 96 well plates or flat bottomed 12 well plates for 12 hours. For cell expansion, the T cell Expansion Kit (Miltenyi) was applied according to the manufacturing protocol. The cytokines IL-2 (20–200 U/l, BD), IL-4 (1–10 pg/ml, BD), IL-6 (500–4000 pg/ml, BD), IL-8 (500–1000 pg/ml, BD), IL-10 (10 ng/ml, BD) and TGF-β (10 ng/ml, BD) were added for 1–7 days.

### Flow cytometry

PDCs were identified as CD123-PE positive, HLA-DR-PerCP positive, lineage cocktail-FITC (CD3, CD14, CD16, CD19, CD20, CD56; Becton Dickinson Heidelberg, Germany) negative and BDCA2-APC/PE (Miltenyi Biotec, Bergisch-Gladbach, Germany) positive cells. Surface antigen staining was performed using the antibodies CD1a-FITC, CD2-PE, CD3-PE, CD4-PERCP, CD4-APC-CY7, CD8-ARC-CY7, CD11c-APC, CD14-APC, CD16-FITC, CD19-FITC, CD20-APC, CD23-PE, CD-25-APC/APC-CY7, CD32-FITC, CD34-PERCP, CD38-APC/PE-CY7, CD40-APC, CD44-PE, CD56-PE, CD64-FITC, CD69-FITC, CD80-FITC, CD83-FITC, CD86-PE, CTLA4-PE, CCR7-PE-CY7, CD117-APC, TCR-α/β-PE/FITC, TCR-γ/δ -APC, IFNg-FITC, IL-4 PE, IL-10 APC (Becton Dickinson Heidelberg, Germany), CD123-APC/FITC, BDCA4-APC, CD45RA-FITC, CD133-PE (Miltenyi Biotec, Bergisch-Gladbach, Germany), CD25-FITC/PE-CY7, CD127-FITC, (eBioscience, San Diego, USA) and GITR-FITC, IL-2 FITC, IL-6-FITC, IL-8-FITC, TGF-β-PE, TNF-α-FITC (R&D Systems, Minneapolis, USA). The Isotype PE IgG1/IgG2a, the Isotype APC IgG1/IgG2a and the Isotype Fitc IgG1/IgG2b (Becton Dickinson, Heidelberg, Germany) were used as negative controls. The Mean Fluorescence Intensity (MFI) was determined separately for each Isotype control and each subpopulation and then subtracted from the positive results before calculating the relative MFI. The figures 1 and 4 show the relative MFI after incubation with HNSCC in relation to the medium control MFI and to the MFI of the classical PDC population (negative for the TCR-αβ), respectively. The control MFI is equal to 1 in each case by definition. Early apoptotic or dead cells were detected by staining the cells with Annexin-V-FITC and Propidium-Iodide-PE (Becton Dickinson, Heidelberg, Germany). Doublets were excluded by gating FSC-A against FSC-H. Only singlets were analysed. CD107a was up-regulated in the PDC population subsequent to stimulation with HNSCC, indicating cytokine secretion.

The amount of 2·10^5^ PDCs was incubated on ice in 50 µl 1% BSA (in PBS) with 1-2 µl of each antibody for 15 minutes. Then the cells were washed three times in 500 µl 1% BSA and subsequently analysed on a FACSCanto (Becton Dickinson) equipped with FACS DIVA software. Intracellular staining was performed with the eBioscience-staining-buffer-set according to the eBioscience protocol. Cell sorting was performed with a Dako moFlow.

### RT-PCR

After cell-sorting and verification of cell purity and viability, mRNA was isolated with the RNeasy kit (Qiagen, Hilden, Germany). For RT-PCR we used the Qiagen One-step RT-PCR kit according to protocol and publications. The primers are listed in table 1. In order to minimize the risk of falsified results by proliferating cells, RT-PCR was performed directly after sorting the PDC population. Stimulated PDCs we isolated at a purity >99%, PDCs in the medium control at a purity >92%.

**Table 1:** Primers for RT-PCR.

PRIMER	FORWARD	REVERSE
NOTCH-1	5′-CGGGGCTAACAAAGATATGC-3′	5′-CCATATGATCCGTGATGTCC-3′ [17]
GATA-3	5′-GCATTCCTCCTCCAGAGTGT-3′	5′-CTCATTAAGCCCAAGCGAAG-3′ [17]
SPI-B	5′-GGCTGTCCAACGGTAAGTCT-3′	5′-GCATACCCCACGGAGAACT-3′ [17]
Pre-TCR-α	5′-GGAGCAGGTCAAACAGCAGC-3′	5′-CCCATCTCTCCCTGCCTTCTG-3′
CD3ε	5′-GGCCTTTCTATTCTTGCTCCAG-3′	5′- GTCTCCATCTCTGGAACCACAG-3′ [47]
CD3γ	5′-ACTCGAATTCCTGAGTTCAATTCCTCCTCAAC-3′	5′-GGAGGAATTCACTGACATGGAACAGGGGAAGG-3′ [48]
CD3δ	5′-GCTGTACTGAGCATCATCTC-3′	5′-GTGAATTGCAATACCAGCATC-3′ [48]
DJ-β	5′-GATCTCATAGAGGATGGTGGC-3′	5′- TGGGAGGGGCTGTTTTTGT-3′ [48]

Subsequent to electrophoresis, the PCR products were sequenced at the University of Kiel.

### Statistical analysis and samples

The Kolmogorow-Smirnow test did not show a normal distribution. Therefore we performed the Wilcoxon-Mann-Withney test for statistical analysis. p<0.05 was considered to be marginally significant. 5–12 samples were analysed per surface marker, cytokine and tissue. Only those FACS plots showing a clear separation between the subpopulations were included. Doublets were excluded by gating FSC-A against FSC-H. Cells were extracted from the blood of healthy donors and metastatic lymph nodes. Solid tumor tissue did not provide PDCs sufficiently for cytokine analysis. Tissue histology was determined by the Institution for Pathology, University of Lübeck.

## Supporting Information

Text S1 Supporting information on the TCR-αβ positive population.
(DOC)

Text S2 An ethic proposal concerning the abstraction of tumor tissue during operations of tumor patients was approved by the ethics committee, Campus Lübeck.
(PDF)

Text S3 Written informed consent was obtained in each case before abstracting tissue and blood.
(PDF)

Text S4 Written informed consent was obtained in each case before abstracting tissue and blood.
(PDF)
