# Supplementary material for: Expression of the T Cell Receptor αβ on a CD123+ BDCA2+ HLA-DR+ Subpopulation in Head and Neck Squamous Cell Carcinoma
Source: PLoS One. 2011 Jan 11;6(1):e15997. doi: 10.1371/journal.pone.0015997 (PMC3019173; doi:10.1371/journal.pone.0015997)
Supplement: Text S2 — An ethic proposal concerning the abstraction of tumor tissue during operations of tumor patients was approved by the ethics committee, Campus Lübeck. (PDF) [file pone.0015997.s002.pdf]

Universität zu Lübeck

Medizinische Fakultät - Der Vorsitzende der Ethikkommission

Dekanat der Medizinischen Fakultät der Universität zu Lübeck
Ratzeburger Allee 160, D-23538 Lübeck

Herrn
Nitsch
Klinik für Hals-, Nasen- und Ohrenheilkunde

im Hause

Bearbeiter: Frau Erdmann
Telefon: (0451) 500- 4639
Fax: (0451) 500- 3026
email: erdmann@zuv.uni-luebeck.de

Datum: 02.05.06
Aktenzeichen:
(immer angeben !) 06-027

nachrichtlich:
Frau Prof. Dr. Wollenberg
Direktor der Klinik für Hals-, Nasen- und Ohrenheilkunde

Sitzung der Ethik-Kommission am 21. März 2006
Antragsteller: Herr Nitsch / Frau Prof. Wollenberg
Titel: Einfluß des Ernährungsstatus auf das lokale inflammatorische Tumormilieu sowie die immunregulatorische Funktion Dendritischer Zellen in Patienten mit Kopf-Hals-Karzinomen

Sehr geehrter Herr Nitsch,

der Antrag wurde unter berufsethischen, medizinisch-wissenschaftlichen und berufsrechtlichen Gesichtspunkten geprüft.

Die Kommission hat keine Bedenken.

Bei Änderung des Studiendesigns sollte der Antrag erneut vorgelegt werden. Über alle schwerwiegenden oder unerwarteten und unerwünschten Ereignisse, die während der Studie auftreten, muß die Kommission umgehend benachrichtigt werden.

Nach Abschluß des Projektes bitte ich um Übersendung eines knappen Schlussberichtes (unter Angabe unseres Aktenzeichens), aus dem der Erfolg/Misserfolg der Studie sowie Angaben darüber, ob die Studie abgebrochen oder geändert bzw. ob Regressansprüche geltend gemacht wurden, ersichtlich sind.

Die ärztliche und juristische Verantwortung des Leiters der klinischen Prüfung und der an der Prüfung teilnehmenden Ärzte bleibt entsprechend der Beratungsfunktion der Ethikkommission durch unsere Stellungnahme unberührt.

Mit freundlichem Gruß und den besten Wünschen für den
weiteren Verlauf Ihrer Forschung bin ich
Ihr

Prof. Dr. med. Dr. phil. H. Raspe
Vorsitzender

anwesende Kommissionsmitglieder: ☒

☒ Prof. Dr. Dr. H.-H. Raspe
(Sozialmedizin, Vorsitzender der EK)
Prof. Dr. F. Hohagen
(Psychiatrie)
Prof. Dr. Dominiak
(Pharmakologie)

Frau H. Müller
(Pflege)
☒ Prof. Wessel
(Kinderchirurgie, Stellv. Vorsitzender der EK)
Herr Dr. Fieber
(Richter am Landgericht Lübeck)
☒ Prof. Schwinger
(Humangenetik)

☒ Herr Prof. Dr. H. L. Fehm
(Medizinische Klinik I)
☒ Frau Prof. Dr. M. Schrader
(Plastische Chirurgie)
☒ Herr Dr. Schultz
(Pädiatrie)
☒ Herr D. Stojan
(Präsident des Amtsgerichtes Lübeck)
