# Supplementary material for: Expression of the T Cell Receptor αβ on a CD123+ BDCA2+ HLA-DR+ Subpopulation in Head and Neck Squamous Cell Carcinoma
Source: PLoS One. 2011 Jan 11;6(1):e15997. doi: 10.1371/journal.pone.0015997 (PMC3019173; doi:10.1371/journal.pone.0015997)
Supplement: Text S3 — Written informed consent was obtained in each case before abstracting tissue and blood. (PDF) [file pone.0015997.s003.pdf]

Universitätsklinikum Schleswig – Holstein
Klinik und Poliklinik für Hals-, Nasen- und Ohrenheilkunde
Campus Lübeck

Direktorin: Prof. Dr. med. Barbara Wollenberg

Informationsblatt für Patienten und Patientinnen zur Teilnahme an der Studie: Wissenschaftliche Untersuchung zur Immuntherapie von Kopf-Halstumoren mit CpG-Oligonukleotiden

1. Was ist der Zweck der Studie?

Aus dem Tumor, sowie dem peripheren Blut sollen Zellen des Immunsystems z.B. plasmazytoide dendritische Zellen (PDCs) isoliert werden. Diese Zellen stellen ein wichtiges Bindeglied in der Immunantwort dar. Über verschiedene Medikamente stimuliert, können sie die Immunantwort und damit die Abwehr des Tumors erheblich beeinflussen.

2. Wie läuft die Studie ab?

Während oder auch vor der chirurgischen Entfernung des Tumors sollen Tumor-, als auch Blutproben, sowie in einzelnen Fällen Leukapherisate gewonnen werden. Aus diesen sollen entsprechende Zellen isoliert werden.

3. Was ist der Nutzen dieser Studie?

Es handelt sich um eine wissenschaftliche Untersuchung. Solche Untersuchungen sind notwendig, um verlässliche, neue medizinische Forschungsergebnisse zu gewinnen. Die Erkenntnisse aus dieser Studie sollen die Immuntherapie bei Kopf-Halstumoren entscheidend fördern und somit die Lebensqualität und Überlebensrate von Patienten mit Kopf-Hals-Tumoren zukünftig verbessern.

4. Gibt es Risiken?

Es gibt keine Risiken.

5. In welcher Weise werden die im Rahmen dieser Studie gesammelten Daten verwendet?

Nur die Prüfer und deren Mitarbeiter, sowie die Gesundheitsbehörde haben Zugang zu den vertraulichen Daten, in denen sie namentlich genannt werden. Diese Personen unterliegen der Schweigepflicht. Die Weitergabe der Daten im In- und Ausland erfolgt ausschließlich zu statistischen Zwecken und Sie werden ausnahmslos darin nicht namentlich genannt. Auch in etwaigen Veröffentlichungen der Daten dieser Arbeit werden Sie nicht namentlich genannt.

6. Entstehen für mich aus der Teilnahme an der Studie zusätzliche Kosten oder Verpflichtungen?

Nein

Für weitere Fragen im Zusammenhang mit dieser wissenschaftlichen Studie stehen wir Ihnen gern zur Verfügung.
