# Supplementary material for: Expression of the T Cell Receptor αβ on a CD123+ BDCA2+ HLA-DR+ Subpopulation in Head and Neck Squamous Cell Carcinoma
Source: PLoS One. 2011 Jan 11;6(1):e15997. doi: 10.1371/journal.pone.0015997 (PMC3019173; doi:10.1371/journal.pone.0015997)
Supplement: Text S4 — Written informed consent was obtained in each case before abstracting tissue and blood. (PDF) [file pone.0015997.s004.pdf]

Universitätsklinikum Schleswig – Holstein
Klinik und Poliklinik für Hals-, Nasen- und Ohrenheilkunde
Campus Lübeck

Direktorin: Prof. Dr. med. Barbara Wollenberg

Einwilligungserklärung zur Teilnahme an der Studie: Wissenschaftliche Untersuchung zur Immuntherapie von Kopf-Halstumoren mit CpG Oligonukleotiden.

Sehr geehrter Patient, sehr geehrte Patientin,

wir laden Sie ein, an der oben genannten Studie teilzunehmen. Die Aufklärung darüber erfolgt in einem ausführlichen Gespräch.

Die Teilnahme an der Studie ist freiwillig und kann ohne Angabe von Gründen durch Sie abgelehnt werden, ohne daß Ihnen hierdurch Nachteile in Ihrer medizinischen Betreuung entstehen.

Diese Studie, das zugehörige Informationsblatt und die Einwilligungserklärung wurden von der zuständigen Ethikkommission geprüft und positiv beurteilt. Bitte unterschreiben Sie die Einwilligungserklärung nur

- wenn Sie Art und Ablauf der Studie vollständig verstanden haben, und
- wenn Sie bereit sind, der Teilnahme zuzustimmen

Ich erkläre mich bereit, freiwillig an dieser Studie teilzunehmen.

Ich bin ausführlich und verständlich über Wesen und Bedeutung dieser Studie aufgeklärt worden. Ich habe darüber hinaus den Text dieser Patientenaufklärung und Einwilligungserklärung, die insgesamt 2 Seiten umfasst, gelesen. Aufgetretene Fragen wurden mir vom aufklärenden Arzt verständlich und genügend beantwortet. Ich hatte ausreichend Zeit, mich zu entscheiden. Ich habe zur Zeit keine weiteren Fragen mehr.

Ich bin zugleich damit einverstanden, daß meine im Rahmen dieser Studie ermittelten Daten aufgezeichnet, ausgewertet und in anonymisierter Form publiziert werden. Eine Kopie der Patienteninformation habe ich erhalten.

____________________ ____________________
Ort, Datum — Name, Unterschrift des Patienten/der Patientin

____________________ ____________________
Ort, Datum — Unterschrift aufklärende/e Ärztin/Arzt
