# Supplementary material for: Expression of the T Cell Receptor αβ on a CD123+ BDCA2+ HLA-DR+ Subpopulation in Head and Neck Squamous Cell Carcinoma
Source: PLoS One. 2011 Jan 11;6(1):e15997. doi: 10.1371/journal.pone.0015997 (PMC3019173; doi:10.1371/journal.pone.0015997)
Supplement: Text S1 — Supporting information on the TCR-αβ positive population. (DOC) [file pone.0015997.s001.doc]

**Reviewer #1:** Thiel et al show a new population of cells positive for PDC markers as well as TCR-<alpha><beta> in within metastatic lymph nodes in patients with HNSCC. This subset of cells indicate an interface between the PDC and T cell lineage produce IL-2, IL-6 and have a D-J-beta rearrangement.

1. A more extensive examination of the cell subset should be performed. For example, are they CD25 positive and can they be expanded using IL-2 or any other cytokines? Are these cells suppressive?

The CD123+ BDCA-2+ HLA-DR+ TCR-αβ+ population was not significantly positive for CD25. Some donors expressed CD25*dim* on a subset of TCR-αβ+ CD123+ BDCA-2+ HLA-DR+ cells. The TCR-αβ+ positive subset was entirely positive for CD25*dim* in single cases. The subset positive for the surface marker CD25 was likewise located in the PDC region (SSC/FSC), however composed of larger cells.

BDCA-2+/CD123+/HLA-DR+

CD25+

CD25-

Expansion of the TCR-αβ+ subset could not be assured, as the TCR-αβ was not detectable after cross-linking CD3/28. Furthermore, we were not able to reach an adequate purity sorting the CD123+ BDCA-2+ HLA-DR+ TCR-αβ+ subset, as they only represented up to 4.5% of the PDC population and even less of the T cell population. The PDC population represented <1% of the metastatic lymph node single cell suspensions. The same has been shown to be significant for peripheral blood cell counts.

Nevertheless, we incubated isolated CD123+ BDCA-2+ HLA-DR+ cells with HNSCC and the T cell Expansion Kit (Miltenyi) in addition to IL-2 (20-200 U/l) for 3-7 days. Less TCR-αβ+ cells were revealed (after 1-7 days) when compared to the control with HNSCC (after 24h).

HNSCC (24h)

HNSCC + Expansion Kit and IL-2 (24h-7days)

Presumably this can be attributed to the down-regulation of the TCR subsequent to cell activation. However, the CD123+ BDCA-2+ HLA-DR+ cells, which usually do not survive longer than 12-24 hours in-vitro, survived up to 7 days subsequent to stimulation with the Expansion Kit and IL-2. This may be associated with proliferation of the TCR-αβ+ subset, however we cannot prove this. Incubation with IL-4 (1-10pg/ml), IL-6 (500-4000pg/ml), IL-8 (500-1000pg/ml), IL-10 (10ng/ml) and TGF-ß (10ng/ml) did not lead to any significant expansion of the subset. Cytokine concentrations were chosen according to the cytokine secretion of the HNSCC cell lines, which induced the TCR-αβ+ subset (19).

Due to the fact that we could not isolate this minor subset with a high purity and even less to a high cell count, it was not possible to analyze the suppressive potency of this population at the time.

Since they also produce TGF-beta, are they positive for FoxP3? 
We were not able to show any expression of FOXP3 and CTLA-4 in the entire CD123+ BDCA-2+ HLA-DR+ population. Furthermore, cross-linking CD3/28, stimulation with IL-2, IL-4, IFN-γ and TGF-β in addition to HNSCC, also failed to induce FOXP3 and CTLA-4 in the entire CD123+ BDCA-2+ HLA-DR+ population.

Is there a Figure 1C, as mentioned in the figure legend to figure 2? 

No, `Figure 1C´ does not exist.

In figure 2C, cells positive for CD123, HLA-DR and BDCA-2 are located in the center of the PDC population when back-gated. I am surprised that no cells fall outside the FSC/SSC gate. Were the TCR positive cells properly back-gated into the whole FSC/SSC or just back into the gated area in 2C? Do any TCR+/CD123+/DR+/BDCA2+ cells end up in any other area outside the gate in fig 2c?

The CD123+ BDCA-2+ HLA-DR+ TCR-αβ+ cells do focus on the PDC location when back-gating them to SSC/FSC. Of course, particular cells fall outside the gate, however they do not seem to present a further population.

BDCA-2 positive cells, back-gated

BDCA-2+/TCR+ cells, back-gated

BDCA-2-/TCR+ cells, back-gated

BDCA-2 +/-, TCR+ cells, back-gated

Change order of top left micrograph in figure 3B. Better align the two bottom graphs of figure 3B. For clarity it would be better to label the lanes in the figure instead of in the legend.

What are the percent-values in figure 5, and are they statistically different compared to "classical PDCs"?

In this particular sample, the TCR-αβ positive subset represented about 2% of the PDC population. As described in the text, this population was enhanced from 1.6% (mean) +/-1.5 % (SD) in the medium control to 4.5% (mean) +/-0.88 % (SD) subsequent to stimulation with HNSCC for 12h. The statistics are presented in figure 6. We did not present the statistics for the percent-values in the previous manuscript, as 90-100% of the TCR-αβ+ cells were positive for IL-2 and 100% of the TCR-αβ+ cells were positive for IL-6 in every sample. Furthermore, the classical PDC do not confidently produce IL-2. The FACS plots show a shift in the population, however no circumscribable positive subset. About 80% of the classical PDCs were positive for IL-6 in every sample (p<0.001).

What was the basis for staining CD5 and CD7?

Different T cell markers (early T cell markers, Treg markers) including CD1a, CD2, CD3, CD4, CD5, CD7, CD8, CD25, CTLA-4, FOX-P3, CD34 and CD117 were analyzed on the TCR-αβ positive subset.

**Reviewer #2:** This manuscript by Dr. Thiel et al. very nicely describes the characteristics of an interesting subpopulation of dendritic cells.  The data is clearly summarized and statistics are well done.  The conclusions are correctly based on the data that are shown. There are only a few very minor comments, as listed below:

- in the pdf file of the manuscript, the scattergrams of the flow cytometric results look blurry.  The data would be much easier to decipher if the clarity of the figures were improved. It is hoped that the jpg or tif versions of the figures would be more clear.

The scattergrams were amended accordingly. In order to improve the quality, we reduced the number of scattergrams.

- Figures 1 and 4:  While the methods section indicates that the MFI for controls is standardized to 1, this should also be indicated in the figure legends to simplify data evaluation by the reader.

The figure legends were amended accordingly.
